# Craniofacial morphology in Apert syndrome: a systematic review and meta-analysis

**DOI:** 10.1038/s41598-022-09764-y

**Published:** 2022-04-05

**Authors:** Mohammad Khursheed Alam, Ahmed Ali Alfawzan, Kumar Chandan Srivastava, Deepti Shrivastava, Kiran Kumar Ganji, Srinivas Munisekhar Manay

**Affiliations:** 1 Orthodontics, Preventive Dentistry Department, College of Dentistry, Jouf University, Sakaka, Saudi Arabia; 2 Department of Preventive Dentistry, College of Dentistry in Ar Rass, Qassim University, Ar Rass, Saudi Arabia; 3 Department of Oral and Maxillofacial Surgery and Diagnostic Sciences, College of Dentistry, Jouf University, Sakaka, Saudi Arabia; 4 Preventive Dentistry Department, College of Dentistry, Jouf University, Sakaka, Saudi Arabia

**Keywords:** Developmental biology, Genetics, Health care, Medical research

## Abstract

This meta-analysis aims to compare Apert syndrome (AS) patients with non-AS populations (not clinically or genetically diagnosed) on craniofacial cephalometric characteristics (CCC) to combine publicly available scientific information while also improving the validity of primary study findings. A comprehensive search was performed in the following databases: PubMed, Google Scholar, Scopus, Medline, and Web of Science, an article published between 1st January 2000 to October 17th, 2021. PRISMA (Preferred Reporting Items for Systematic Reviews and Meta-Analyses) guidelines were followed to carry out this systematic review. We used the PECO system to classify people with AS based on whether or not they had distinctive CCC compared to the non-AS population. Following are some examples of how PECO has been used: People with AS are labeled P; clinical or genetic diagnosis of AS is labeled E; individuals without AS are labeled C; CCC of AS are labeled O. Using the Newcastle–Ottawa Quality-Assessment-Scale, independent reviewers assessed the articles' methodological quality and extracted data. 13 studies were included in the systematic review. 8 out of 13 studies were score 7–8 in NOS scale, which indicated that most of the studies were medium to high qualities. Six case–control studies were analyzed for meta-analysis. Due to the wide range of variability in CCC, we were only able to include data from at least three previous studies. There was a statistically significant difference in N-S-PP (*I*^2^: 76.56%; *P* = 0.014; CI 1.27 to − 0.28) and Greater wing angle (*I*^2^: 79.07%; *P* = 0.008; CI 3.07–1.17) between AS and control subjects. Cleft palate, anterior open bite, crowding in the upper jaw, and hypodontia occurred more frequently among AS patients. Significant shortening of the mandibular width, height and length is the most reported feature in AS patients. CT scans can help patients with AS decide whether to pursue orthodontic treatment alone or to have their mouth surgically expanded. The role of well-informed orthodontic and maxillofacial practitioners is critical in preventing and rehabilitating oral health issues.

## Introduction

It's estimated that only 4.5% of people with craniosynostosis have Apert syndrome (AS), which is also known as acrocephalosyndactyly^1^. Baumgartner and Wheaton were the first to describe AS clinically in 1842, and Eugene Apert, a French pediatrician, published a series of cases in 1906, in which he reviewed the condition extensively^2^. The syndrome affects anywhere from 1/65,000 to 1/200,000 newborns, regardless of gender^3,4^. In nearly all cases, missense mutations in the FGFR2 gene (on chromosome 10q25–10q26) are to blame, and these are only found in men^5–7^. People with AS are 50% more likely to have a child with AS, according to the research that has been done on the subject of passing the syndrome on to future generations^8,9^. In AS, fibroblasts are unable to produce the essential fibrous material found in several craniofacial tissues, such as bone sutures and cartilage, as well as during tooth formation and regeneration due to the FGFR family of mitogenic signaling molecules (FGFRs)^10^. The mutated FGFR2 gene may therefore have an impact on the dental abnormalities seen in AS^11^.

Most patients with AS have long, lean heads with high foreheads and sunken eyes, as well as abnormalities in the way their eyelids close. This is due to the disordered growth of the skull and face^12^. AS is associated with several other health problems, including a lack of intellectual development, obstructive sleep apnea, and frequent ear infections^13^. Craniosynostosis, hypoplasia of the midface, and syndactyly of the hands and feet are other phenotypic features of AS^14^. In addition, AS is associated with a wide range of significant central nervous system abnormalities, which may be due to the prevalence of mental deficiency in patients with this syndrome^15^. Patients with concave facial profiles may have a reduced volume of nasopharyngeal and oropharyngeal spaces due to hypoplasia of the middle third of the face. Chronic mouth breathing, breathing difficulties, or even sudden death can result from the combination of this and possible posterior nasal stenoses^16,17^. Dental crowding, skeletal anterior open bite, unilateral crossbite, Angle Class III malocclusion, lip with inadequate posture, cleft palate, uvula Bifida in 30% of the palates, upper and lower palate, macroglossia, retained teeth, and thick gums are all symptoms of AS in the mouths of those diagnosed with the condition^18,19^.

However, until now, no systematic review of the existing literature has been conducted on the CCC of AS patients. This meta-analysis aims to compare AS patients with non-AS populations on CCC to combine publicly available scientific information while also improving the validity of primary study findings. When it came to CCC obtained from lateral teleradiographs or computed tomography (CT) scans of the head, there was no difference between people with AS and the general / non-AS population, according to the review.

## Materials and method

### Search strategy

**Figure 1:**
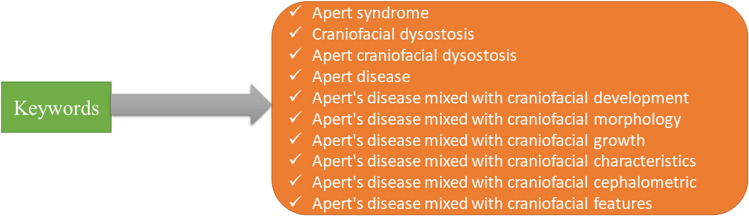
Keywords used in search strategies.

**Figure 2:**
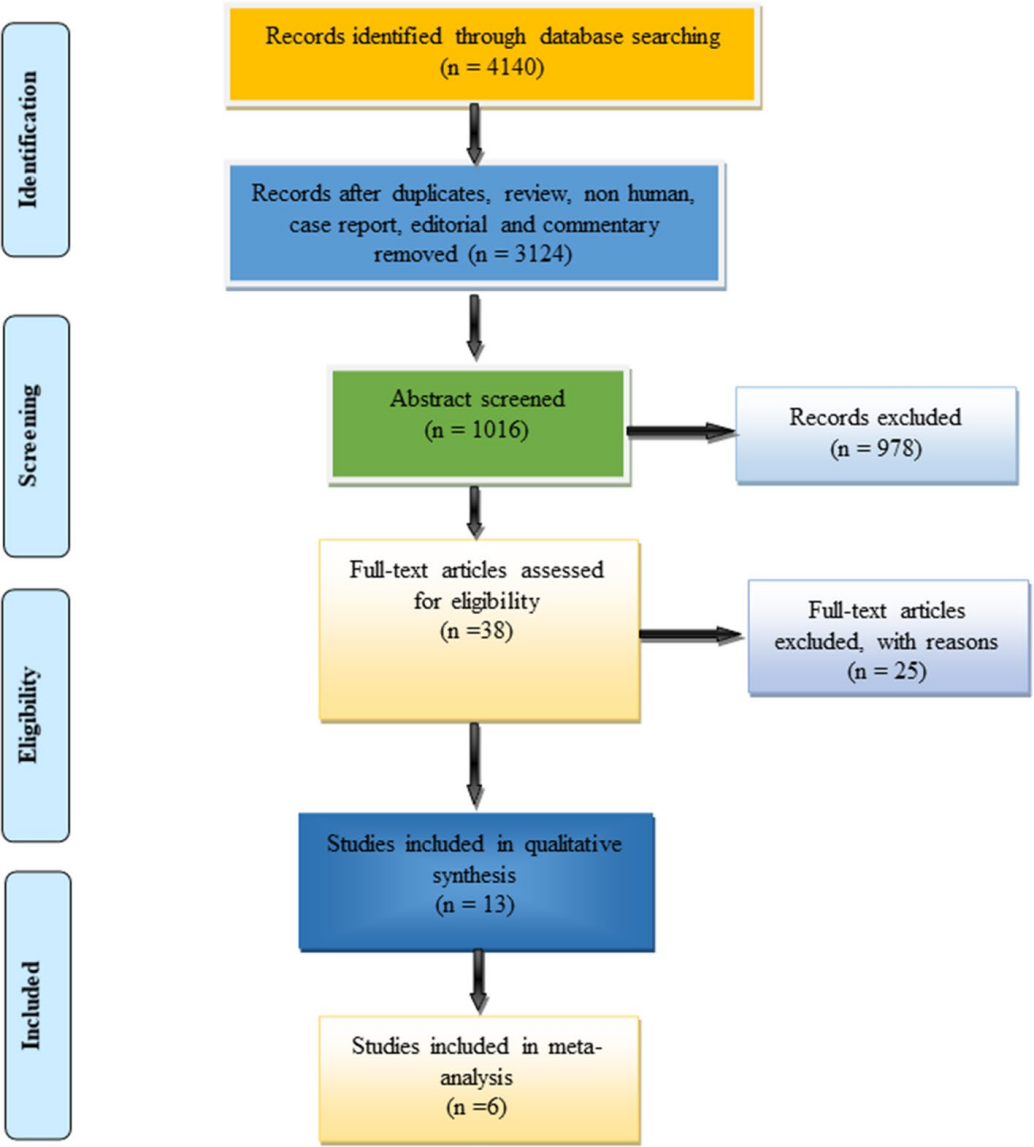
PRISMA flow diagram of the search strategies.

The following databases were searched thoroughly: PubMed, Google Scholar, Scopus, Medline, and Web of Science, with restrictions on the publication date from 1st January 2000 to October 17th, 2021. The keyword combinations were used mixed with the Boolean operator “AND” (Fig. 1). Search includes peer-reviewed journals that have published full-text articles on the AS being discussed, as well as articles written in English and published there. Among the types of research that have been ruled out are animal studies, clinical case reports, pilot studies, bibliographic reviews, systematic reviews, and chapters from full-length books. Figure 2 depicts the process of selecting the articles, which consisted of four steps. This systematic review was conducted followed by the PRISMA^20^ (Preferred Reporting Items for Systematic Reviews and Meta-Analyses) guidelines and registered at the University of York's (U.K.) PROSPERO database (Registration No. CRD42021282637).

### Study selection criteria

We used the PECO^21^ system to classify people with AS based on whether or not they had distinctive CCC compared to the non-AS population. Following are some examples of how PECO has been used: People with AS are labeled P; clinical or genetic diagnosis of AS is labeled E; individuals without AS (not clinically or genetically diagnosed) are labeled C; CCC of AS are labeled O and are examined using lateral cephalometric measurements or a CT scan. There were case–control, cross-sectional, and cohort studies that compared the Cephalometric and CT scan measurements of people with AS to non-AS participants that met the inclusion criteria. Two researchers (MKA & KCS) worked independently on the selection process, and any discrepancies over the results were resolved by consensus. There was a third assessor called in when the first two couldn't agree (DS). There was also a manual search of the papers' bibliographic references that were discovered during the initial search.

### Data extraction and quality assessment:

**Figure 3:**
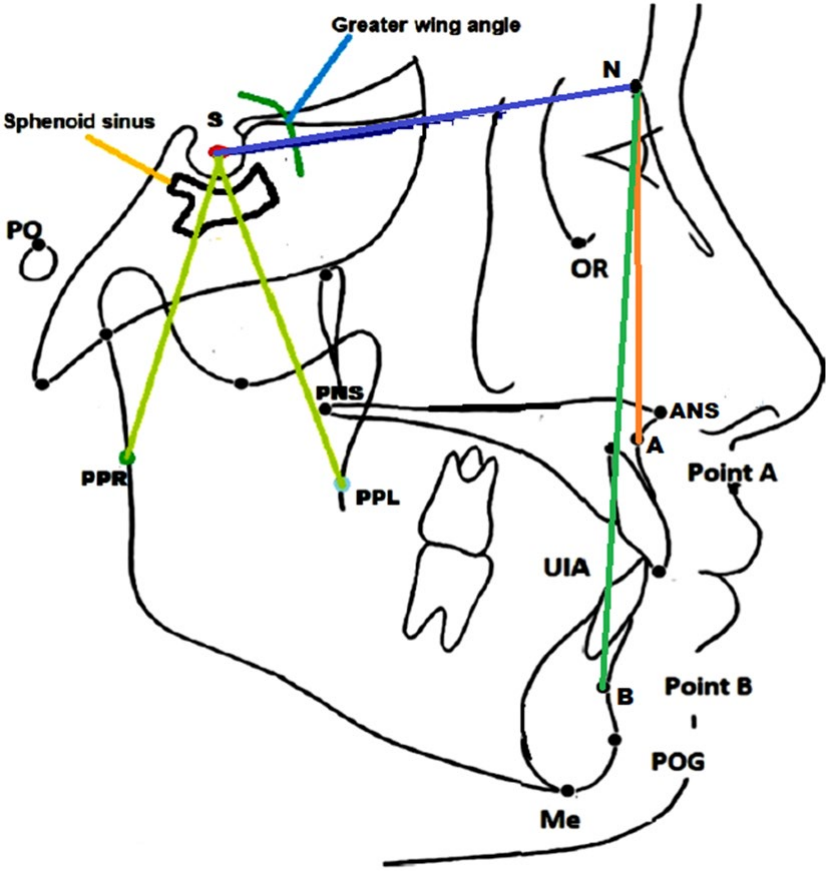
Cephalometric landmark of the different angle.

Two researchers (MKA and KCS) retrieved the following information from each article: authors with year, country, sample size, gender, and final remarks from the study. Given the wide range of CCC used in the papers, it was decided that only those measurements that were replicated in at least two articles would be included in the meta-analysis, those were: SNA°, SNB°, PPR-S-PPL, N-S-PP and greater wing angle (Table 1 and Fig. 3). For each measurement, the mean value and standard deviation were recorded. To determine the methodological quality of the papers, three examiners used the Newcastle–Ottawa Quality Assessment Scale (NOS)^22^: two working collaboratively (MKA and KCS) and a third working independently (DS).

**Table 1:** Cephalometric landmark with their definitions.

SNA angle	A measurement of the maxilla's anteroposterior distance from the cranial base
SNB angle	The angle between the Sella/Nasion plane and the Nasion/B plan
Greater wing angle	Measured from the plane pass the midpoint of bilateral most in front points of corneas and parallel to Frankfort horizontal plane, indicates the sphenoid greater wing divergence
PPR-S-PPL	The angle between bilateral lateral pterygoid plates, measured by connecting points PPR, S, and PPL, indicates the separation of lateral pterygoid
N-S-PP	Angle between N, S, and PP, corresponding to the degree of backward rotation of the pterygoid plates

### Statistical analysis

Each finding was the subject of a meta-analysis of its own. There could only be a meta-analysis when a mean datum was presented in at least three articles because the CCC taken by different publications differed. As expected, there was some evidence of heterogeneity among the individual studies, so a random-effects model was used. Each outcome was assigned a pooled effect size (mean difference) and a 95 percent confidence interval. Heterogeneity in effect size was examined with the Q statistic, I^2^ index with Galbraith plot. A statistically significant Q statistic result (*p* 0.05) revealed heterogeneity. Furthermore, indices of heterogeneity I^2^ of approximately 25%, 50%, and 75% were found to indicate low, moderate, and significant heterogeneity, according to the results. All statistical analyses were carried out using MedCalc (version: 19.3) and R studio (metafor package).

## Results

### Selection of studies

Our initial search strategy yielded 4140 papers from databases such as PubMed, Web of Science, Google Scholar, Scopus, and ScienceDirect. After eliminating 3124 papers in the detection phase, the remaining 1016 papers were further screened (review, summary documents, non-human, editorials, case reports, commentaries, letters, and duplicate studies). A total of 38 studies were considered worthy, but 25 were excluded due to unusable data formats. Thus, based on the research objectives and inclusion and exclusion criteria, 13 studies were eventually included in this study (Fig. 2), and the full text of all included studies was retrieved. Only six studies were included in the meta-analysis synthesis.

### Study characteristics

Table 2 summarizes the key characteristics of the included studies. All of the studies included were published in peer-reviewed journals. Six of the 13 studies were conducted in the United States^23–28^, three in Netherlands^29–31^, and one in France^32^, Brazil^33^, Japan^34^ and Italy^35^. The most common method used in the studies was cephalometric measurement. In total, 225 cases were included from all studies, compared to 1688 controls. Boutros^28^ reported the fewest cases (2), while Lu^24^ reported the most cases^33^. Cleft of the soft palate, anterior open bite, severe crowding in the maxillary dental arch, and congenitally missing teeth occurred more frequently among AS patients^34^. Significant shortening of the mandibular width and length, and, subsequently, reduced height is the most reported feature in AS patients^24,27,28,32^.

**Table 2:** Characteristics of the study included in the systematic review.

No	References	Country	Study design	participants	Age range (years)	Sex (M/F)	Method	Craniofacial findings
1	Kobayashi et al.^34^	Japan	Cohort	Apert—7 Comparator—12	Case: 12.3(5)* Comparator: 10.8(2.89)*	Case 4/3 Comparator: 6/6	Cephalometric analysis	AS patients had significantly more severe maxillary hypoplasia in two dimensions and increased clockwise mandibular rotation
								Cleft of the soft palate, anterior open bite, severe crowding in the maxillary dental arch, and congenitally missing teeth occurred more frequently among AS patients
2	Lu et al.^23^	U.S.A	Case–control	Apert—25 Control—20	Case: 2 days to 6 years Control: 4 days to 16 years	Apert: 12/13 Control: 9/11	CT scan with Cephalometric landmark	N-S-BA and N-SO-BA angles of AS were more narrowed compared to normal
3	Meazzini et al.^35^	Italy	Cohort	Apert—19 Control—38	1–12 (age matched)	Case: N/A Control: 20/18	CT scan	AS showed a significant earlier ossification of all sutures compared to the nonsyndromic group
								Care should be taken when planning any maxillary orthopedics, such as expansion or maxillary protraction, given the high frequency of early fusion of circummaxillary sutures
4	Morice et al.^32^	France	Case–control	Apert—12 Control—12	8.9(9.2)* (age matched)	Case—6/6 Control: matched	CT scan with DICOM raw data using 3D Slicer	Open gonial angle, short ramus height, and high and prominent symphysis
								Short ramus height appeared more pronounced in Apert than in Crouzon syndrome
5	Lu et al.^26^	U.S.A	Case–control	Apert—33 Control—54	0–62 (age matched)	Case: 18/15 Control: 29/25	CT scan with Cephalometric analysis	Initially significant shortening of the mandibular width and length, and, subsequently, reduced height
								Apert has less shortening in mandibular height with the more shortened posterior cranial base length
								Limited nasopharyngeal and oropharyngeal airway space
6	Lu et al.^24^	U.S.A	Case–control	Apert—18 Control 36	Case: 4 days to 24 years Control: 5 days to 24 years	Apert: 10/8 Control: 22/14	CT scan with Cephalometric landmark	The zygoma markedly retruded
								Maxillary anterior posterior dimension was 22% shorter than normal, transverse width of the zygoma increased 39% between 6 months and 2 years of age
7	Lu et al.^25^	U.S.A	Cohort	Apert—18 Control—36	0–24 (age matched)	Case:10/8 Control:22/14	CT scan with Cephalometric analysis	The angulation changes occur earlier in development, than linear distance deformity (largely shortening) in AS patients compared with controls
								The initial facial deformity of AS occurs in maxilla, while the orbit deformity develops later
8	Forte et al.^33^	Brazil	Case–control	Apert—19 Control—17	6–13(age matched)	N/A	Cephalometric analysis	Midface retrusion associated with altered sphenoid morphology (widened and retruded pterygoid plates)
								A flatter and wider maxilla, suggesting diminished growth inferiorly and anteriorly
9	Reitsma et al.^30^	Netherlands	Population based Case–control l	Apert—28 Control—451	Case—3.9–15.1 Control—N/A	Case: 10/18 Control: 225/226	Panoramic radiographs	Girls with AS had a statistically significant less mature dental maturity compared with controls
								Dental maturation was more delayed than control
10	Reitsma et al.^29^	Netherlands	Population based Case–control	Apert—7 Control—486	Case: 12–19 years Control: 4–22	Case—0/7 Control—N/A	Cephalometric analysis	The SNA, ANB, and SN/PP angles were significantly smaller in the syndromic patients, and the LFH ratio was significantly larger than control values
11	Reitsma et al.^31^	Netherlands	Population based Case–control	Apert—28 Control—457	4-14 years (age matched)	Case—12/16 Control—216/241	CT scan with Cephalometric analysis	Maxillary intercanine width for patients with AS were increased, whilst other arch width variables showed no change
								Dental arch dimensions were found to be consistently smaller with a diminished growth potential
12	Wink et al.^27^	U.S.A	Case–control	Apert—9 Control—9	Case: 12–17 Control: 1–18	Case—4/5 Control—5/4	CT scan with Cephalometric analysis	The mandible deformities in the population with AS are likely to be secondary to maxillary hypoplasia and, possibly, the degree of advancement and end point position from maxillary growth
13	Boutros et al.^28^	U.S.A	Case–control	Apert—2 Control—60	5–15 (age matched)	Case—N/A Control-30/30	Cephalometric analysis	Significant reduction in bicondylar width compared with normal
								The ramus appears torqued inward, forming a greater angle with the cranial base

*N/A* not available.

*Mean (SD).

### Meta-analysis

**Figure 4:**
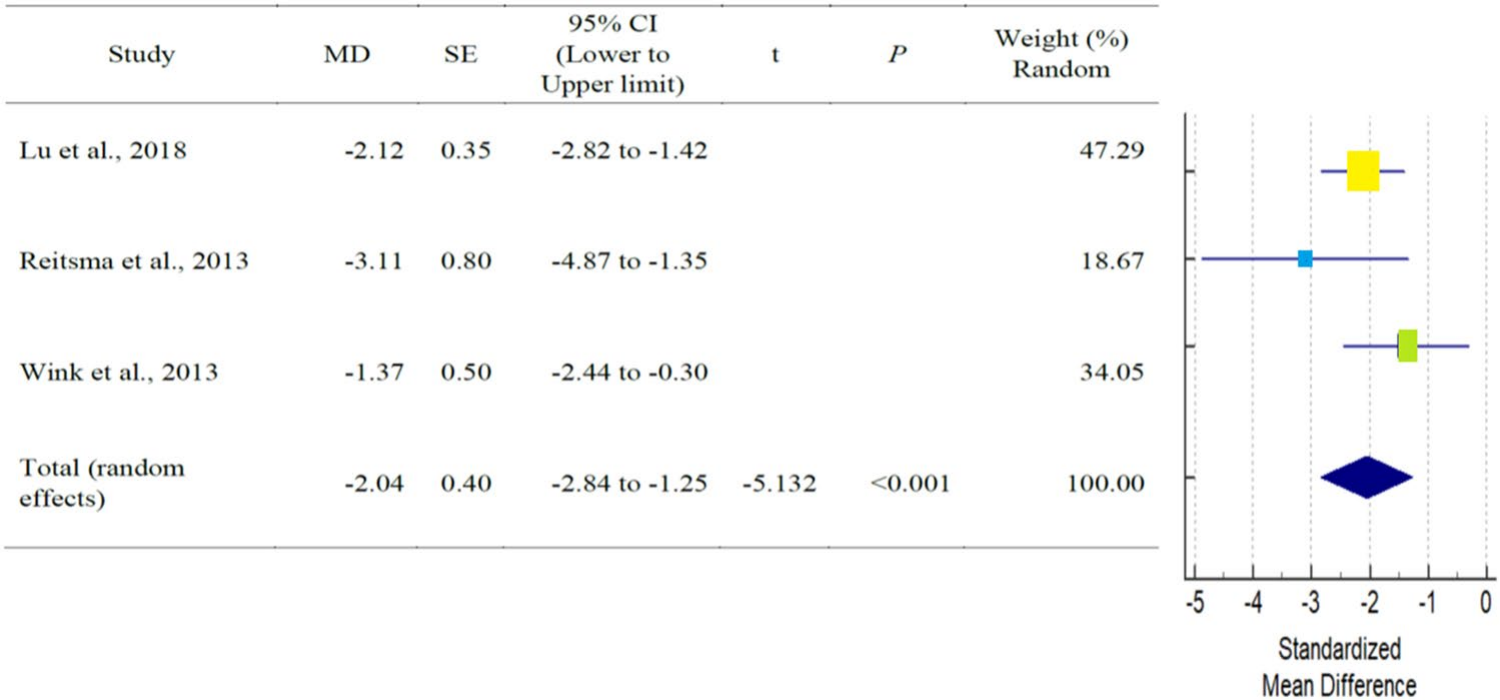
Forest plot for the outcome of SNA.

**Figure 5:**
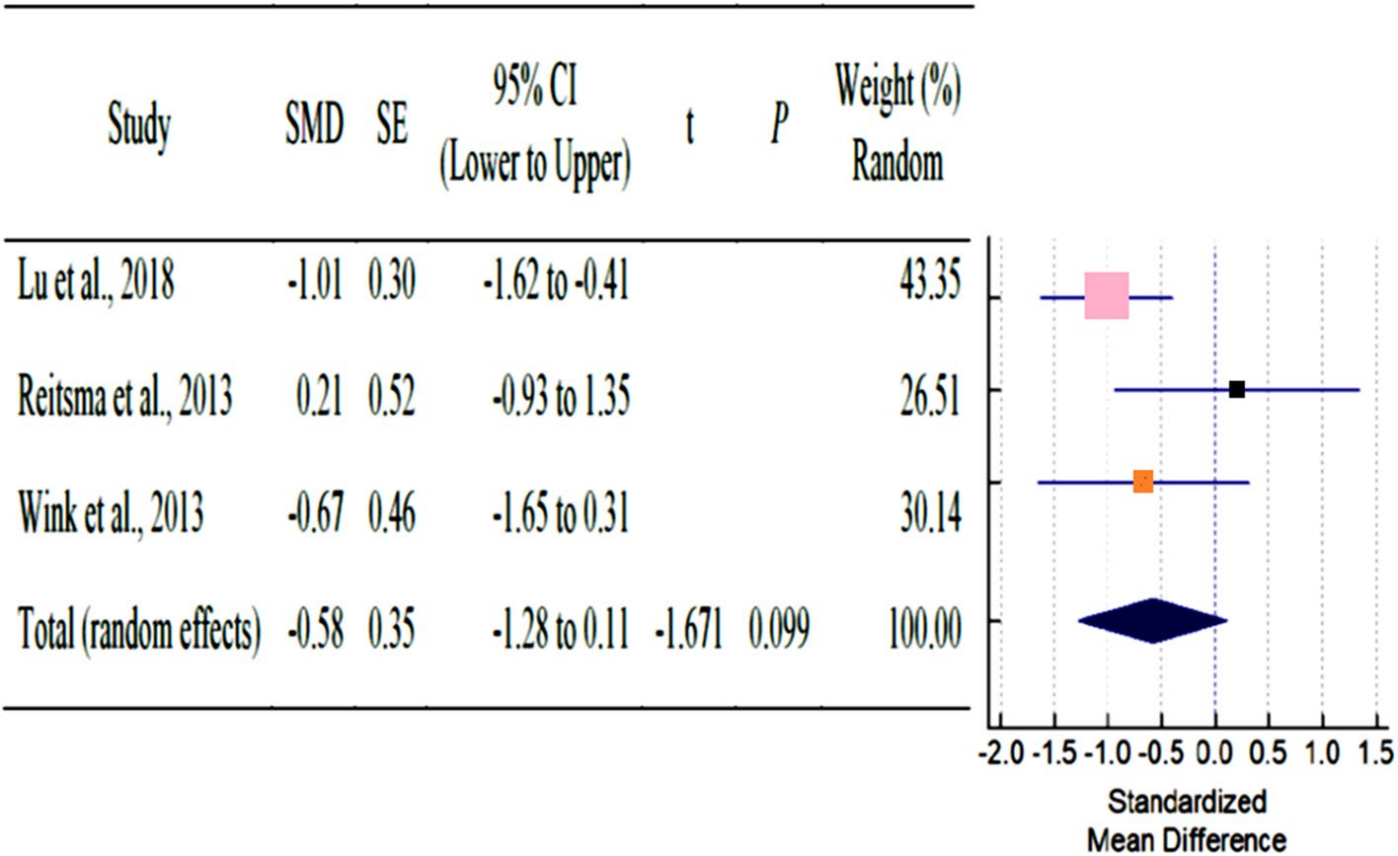
Forest plot for the outcome of SNB.

**Figure 6:**
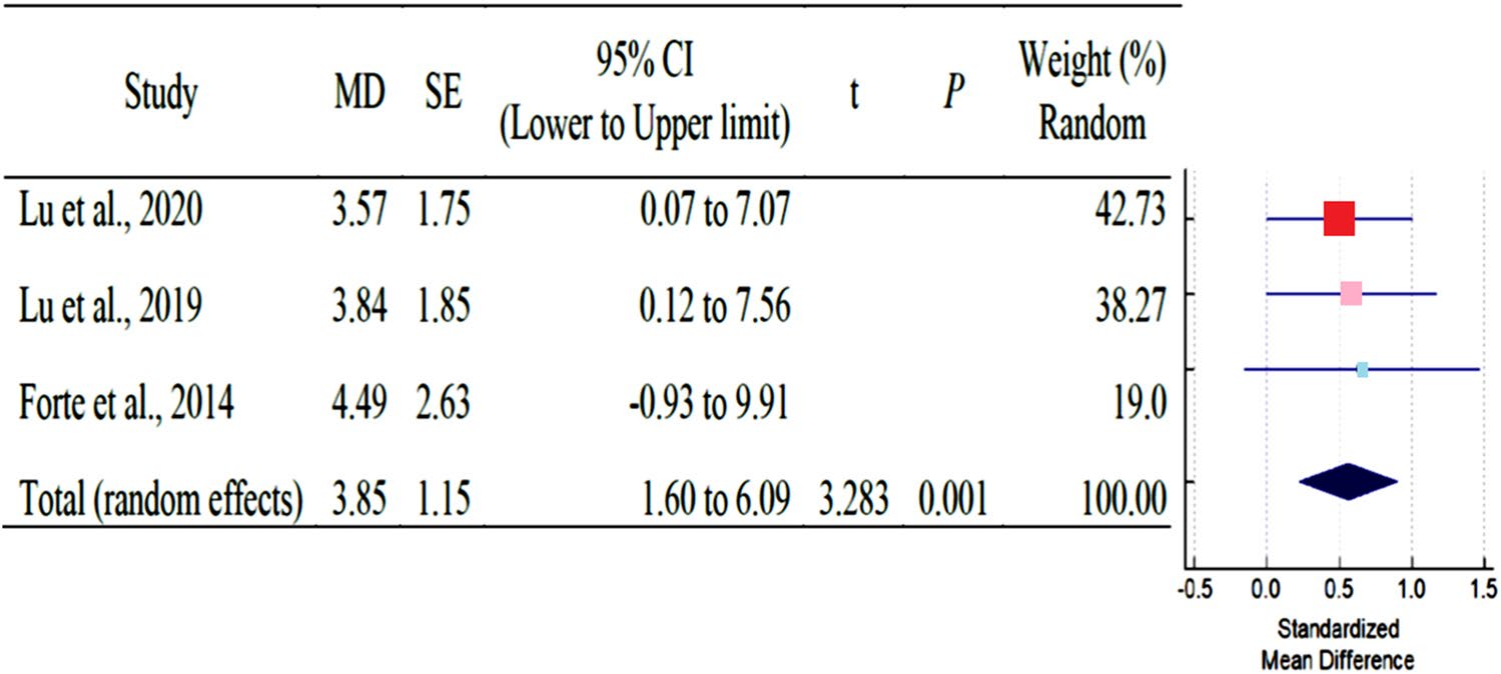
Forest plot for the outcome of PPR-S-PPL.

**Figure 7:**
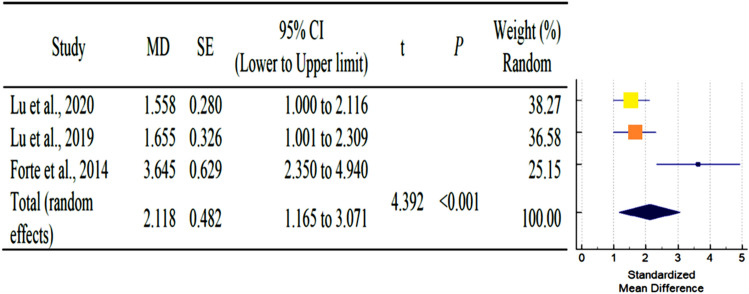
Forest plot for the outcome of N-S-PP.

**Figure 8:**
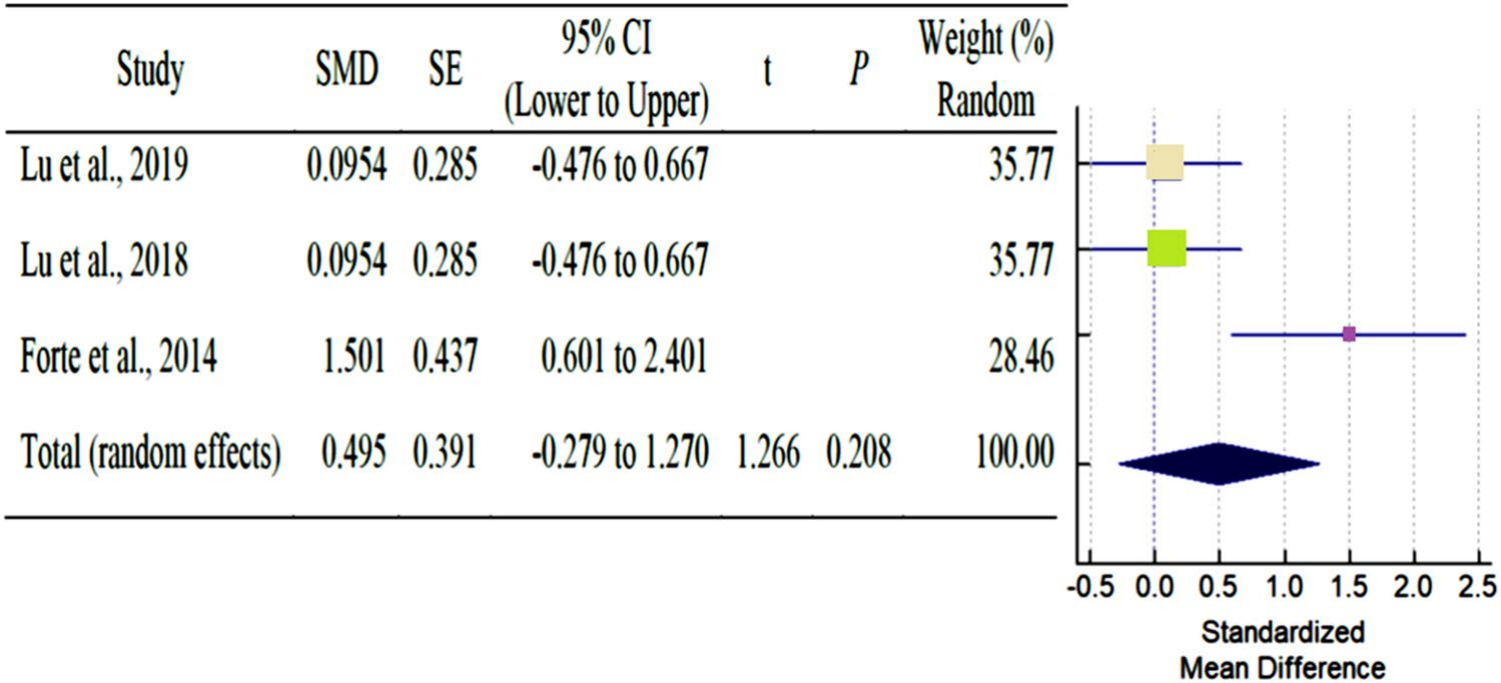
Forest plot for the outcome of SNA greater wing angle.

**Figure 9:**
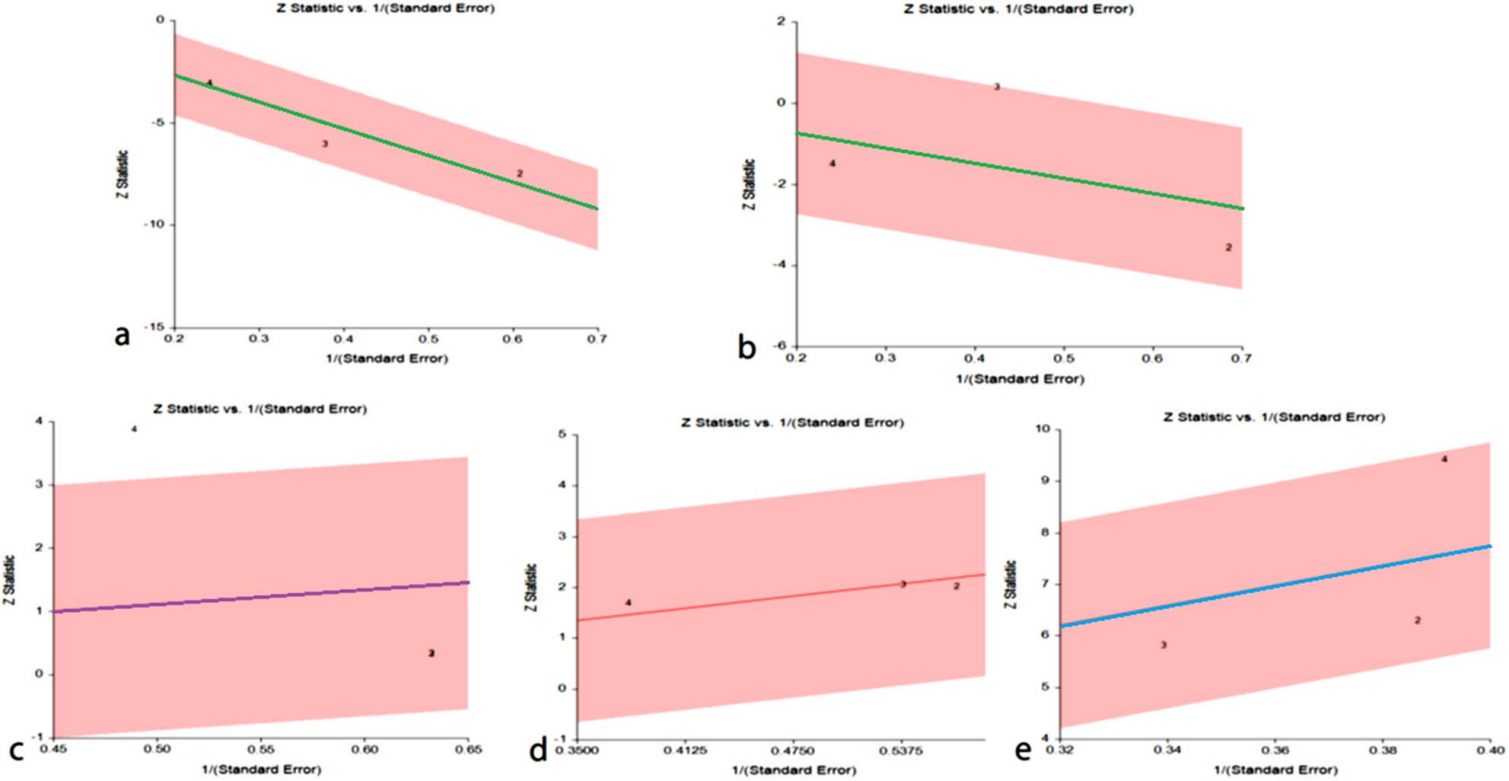
Galbraith plot for the outcome of (**a**) SNA, (**b**) SNB, (**c**) PPR-S-PPL, (**d**) N-S-PP, and (**e**) greater wing angle.

As shown in Table 3, a total of six studies reported outcomes. Except for SNA, and SNB angle of AS subjects had fewer mean outcomes than non-AS subjects. There was a statistically significant difference in N-S-PP and Greater wing angle between AS and non-AS subjects. Because there were so few studies, statistical power issues necessitate a cautious approach to interpreting Q statistics. The I^2^ index provides a more accurate way of assessing effect size heterogeneity. Table 3 showed all pooled mean differences of the individual outcome. Forest plot and Galbraith plot were generated to visualize the heterogenicity of the individual studies. Only N-S-PP and Greater wing angle outcomes showed high heterogeneity, as shown in Table 3. Subgroup analyses were performed for each outcome to assess the potential, differences in effect sizes. Effect size and heterogenicity of the SNA° (*I*^2^: 44.49%; *P* = 0.165; CI 1.26 to − 2.84) (Fig. 4), SNB° (*I*^2^: 52.00%; *P* = 0.124; CI 0.11 to − 1.28) (Fig. 5), PPR-S-PPL (*I*^2^: 11.34%; *P* = 0.939; CI 6.09–1.60) (Fig. 6), N-S-PP (*I*^2^:76.56%; *P* = 0.014; CI:1.27 to -0.28) (Fig. 7), and Greater wing angle (*I*^2^: 79.07%; *P* = 0.008; CI 3.07–1.17) (Fig. 8) were shown by Forest plot and Galbraith plot (Fig. 9). The PPR-S-PPL point has the greatest mean difference when compared to non-AS patients (MD = 3.48). Both SNA (MD = − 2.05) and SNB (MD = − 0.58) angle were short compared to non-AS. AS patients had wider greater wing angle (MD = 2.12, *P* = 0.008) with a significant difference to the non-AS patients.

**Table 3:** Pooled mean differences and heterogenicity of each outcome.

Outcome	Mean differences	95% CI		*Q*	*df*	*p*	*I*^2^ (%)
		Upper limit	Lower limit				
SNA	− 2.05	1.26	− 2.84	3.60	2	0.165	44.49
SNB	− 0.58	0.11	− 1.28	4.17	2	0.124	52.00
PPR-S-PPL	3.48	1.60	6.09	0.09	2	0.939	11.34
N-S-PP	0.50	1.27	− 0.28	8.53	2	0.014	76.56
Greater wing angle	2.12	1.17	3.07	9.66	2	0.008	79.07

*CI* confidence interval.

### Risk of bias

**Figure 10:**
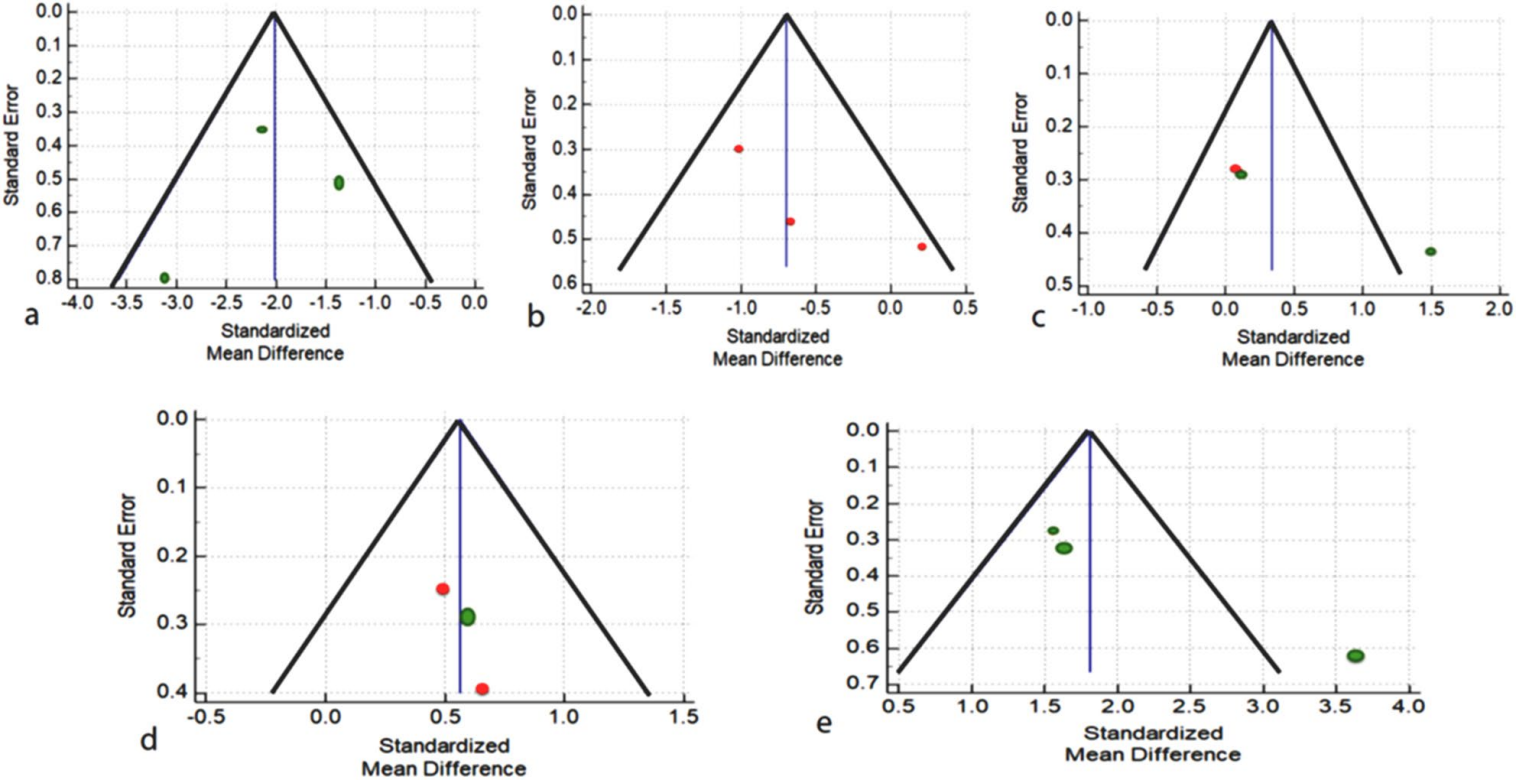
Funnel plot for the outcome of (**a**) SNA, (**b**) SNB, (**c**) PPR-S-PPL, (**d**) N-S-PP, and (**e**) greater wing angle.

NOS was used to find the risk of bias in the included studies (Table 4) (Wells et al*.*2019). Four of the thirteen items received a maximum score of eight, with the remaining four receiving a score of seven. A funnel plot was generated to visualize publication bias among the studies (Fig. 10). It illustrates the relationship between the included studies' effect estimates and their precision or study size. Asymmetry in the funnel plot indicates a lack of homogeneity and reporting bias. In addition, asymmetry can be caused by poor methodological design and studies with small sample sizes. In addition to the reasons, language bias (English language only) and citation bias may also contribute to the asymmetry.

**Table 4:** Methodological quality assessment of the studies by Newcastle–Ottawa quality scale assessment (NOS).

References	Selection			Comparability			Exposure			Total score
	1	2	3	4	5	6	7	8	9	
Kobayashi et al.^34^	*	*	*	*	*	*	*	*	–	8
Lu et al.^23^	*	*	*	*	*	*	*	*	–	8
Meazzini et al.^35^	*	*	–	*	*	*	–	*	–	6
Morice et al.^32^	*	*	–	*	*	*	–	*	–	6
Lu et al.^26^	*	*	*	*	*	*	*	*	–	8
Lu et al.^24^	*	*	*	*	*	*	*	*	–	8
Lu et al.^25^	*	*	–	*	*	*	*	*	–	7
Forte et al.^33^	*	*	*	–	*	*	*	*	–	7
Reitsma et al.^30^	*	*	–	*	*	*	–	*	–	6
Reitsma et al.^29^	*	*	–	*	*	*	–	*	–	6
Reitsma et al.^31^	*	*	–	*	*	*	*	*	–	7
Wink et al.^27^	*	*	–	*	*	*	*	*	–	7
Boutros et al.^28^	*	*	*	*	*	–	–	*	–	6

1—Adequate case definition; 2—Representativeness of the cases; 3—Selections of control/comparator; 4—Definitions of control/comparator; 5—case; 6—Control/ comparator; 7—Exposure of evaluation; 8—Same method for case and control; 9—Nonresponse rate.

## Discussion

Meta-analysis was performed to compare the CCC of AS patients to that of non-AS patients in the population in general. When conducting the literature search, it was decided that all English-language articles would be included within the specific period, ensuring that no relevant information would be omitted from consideration. When conducting a meta-analysis, only measurements that were repeated at least three times were used; therefore, only those measurements were included in the analysis. When interpreting the results of this meta-analysis, caution should be taken.

There is an identifiable genetic cause for the early closure of the cranial sutures in patients with AS. Mutations on Chromosome 10 (q25–10q26) in the gene for FGFR2 are responsible^16^. The majority of these mutations are caused by the substitution of a nitrogen base, which results in an increase in function^36^. AS is passed down in an autosomal dominant manner due to mutations that cause a gain of function.

Compared to non-AS patients, AS patients' dental development was delayed^30^. Children with AS had smaller arch dimensions than other children. From primary to mixed dentition, the dimensions of the dental arch hardly changed^31^. Increased mandibular asymmetry, increased lower facial height ratios, decreased transverse dimensions, an increased inclination of the palatal plane, and a more protruding mandible were observed in the AS patients compared to non-AS. Patients with AS had a more severe abnormal craniofacial growth pattern, morphology, and mandibular asymmetry compared to patients with another craniosynostosis^37^.

SNA and SNB angles were found to be smaller in AS patients, but the anteroposterior position of the maxilla was found to be the same in both AS and non-AS^6,25,27,29^. CCC of the mandible and maxilla can be misinterpreted both anteroposteriorly and vertically if a person has an abnormal cranial base. As a result, many researchers advise taking CCC with the Frankfort plane as a Ref.^38^. A reduced anterior cranial base, despite AS's smaller maxilla, can make the SNA similar to that of controls because the N point would be located further posteriorly^23,35^.

Midface retrusion is influenced by the pterygoid plates' posterior/counterclockwise rotation and their articulation with the maxilla. The pterygoid plate is rotated posteriorly, resulting in the retrusion of the maxilla and the entire midface^33,39^. The sphenoid growth center is a responsible and explanatory variable for the deformity of both the maxilla and the sphenoid. The maxilla was held up or pulled back in space by the sphenoid when the pterygoid plates were rotated posteriorly, pointing to the sphenoid as the primary culprit. As the right and left greater wings diverge more sharply, the orbital contents are pushed forward and the orbital cavity is reduced in size^33,40,41^.

In AS, synchondrosis of the upper and lower jaw occurs in the same time, between the ages of 2 and 6^23,42^. Syndromes may immobilize their midline cranial base due to the advanced closure of the Sphenooccipital and Ethmosphenoid synchondroses^23^. The development of the midface in a transverse direction is likely to be disproportionately compounded. Maxillary width is affected by AS, with an increase in the greater wing angle and the separation of the bilateral lateral pterygoid plates. Maxillary growth does not always follow the regulation of the cranial base, and it may be influenced by the regulatory and masticatory functions of the maxillary bone itself. Rather than relying solely on the skull base, a 'double adjustment' mechanism may also influence the zygoma's growth pattern^24,25^.

It was possible to abandon the idea that there were no significant differences in CCC between people with AS and the general population, even though this study had several flaws, although the significant difference was discovered. These CCC must be combined, and reference planes used to determine the different positions of the maxilla and mandible to arrive at conclusive findings^43^. With so few studies reporting on each outcome metric, it is critical to proceed with extreme caution when interpreting the results. A meta-analysis may yield a different result if more studies are included because of the small number of research studies included.

## Conclusion

The cranial base lengths of AS patients were reduced in proportion to the severity of the disease. In early infancy, the zygoma is the most severely deformed anatomical facial structure, in positional relationships and geometrical shape. In AS person's midface is protruding and, the mandible points down, the orbital volume is smaller, hypoplasia, a delay in dental development, open bites with dental crowding, and cleft palates. CT scans can help patients with AS decide whether to pursue orthodontic treatment alone or to have their mouth surgically expanded. The role of well-informed orthodontic and maxillofacial practitioners is critical in preventing and rehabilitating oral health issues.
